# The effects of *Dendrobium* species on the metabolic syndrome: A review

**DOI:** 10.22038/IJBMS.2023.65997.14512

**Published:** 2023

**Authors:** Zahra Oskouei, Mahboobeh Ghasemzadeh Rahbardar, Hossein Hosseinzadeh

**Affiliations:** 1 Department of Pharmacodynamics and Toxicology, School of Pharmacy, Mashhad University of Medical Sciences, Mashhad, Iran; 2 Pharmaceutical Research Center, Pharmaceutical Technology Institute, Mashhad University of Medical Sciences, Mashhad, Iran

**Keywords:** Dendrobium, Diabetes, Dyslipidemia, Hypertension, Metabolic syndrome

## Abstract

Metabolic syndrome (MetS) is known as a global health challenge with different types of health conditions such as hypertension, hyperglycemia, the increasing prevalence of obesity, and hyperlipidemia. Despite much recent scientific progress, the use of traditional herbal medicines with fewer side effects is increasing worldwide. *Dendrobium*, the second-largest orchid genus, has been used as a natural source of drugs for the treatment of MetS. The beneficial effects of *Dendrobium*, including anti-hypertension, anti-hyperglycemia, anti-obesity, and anti-hyperlipidemic against MetS have been shown in the scientific evidence. The anti-oxidant and lipid-lowering effects of *Dendrobium* modulate hyperlipidemia via reducing lipid accumulation and maintaining lipid metabolism. Restoring pancreatic beta cells and regulating the insulin signaling pathway are involved in its antidiabetic properties. The hypotensive effects contribute to increasing nitric oxide (NO) generation and inhibiting extracellular signal-regulated kinase (ERK) signaling. More research projects, especially clinical trials, are needed to investigate the safety, efficacy, and pharmacokinetics of *Dendrobium* in patients. This review article provides, for the first time, comprehensive information about the efficacy of different species of *Dendrobium*. The described species can be a source of medicines for the treatment of MetS, which are reported in various evidence.

## Discussion

Nowadays, metabolic syndrome (MetS) affects more than a billion people worldwide (1) and in both developed and developing countries, some factors, including industrial lifestyle, unhealthy diet, and high levels of stress have resulted in a higher prevalence of MetS (2). MetS is a serious problem defined as a group of disorders including hypertension (3), obesity, hyperlipidemia (4), insulin resistance, glucose intolerance (5), fatty liver (6), low levels of high-density lipoprotein (HDL), and high blood amounts of triglycerides (TG) (7), which increases the prevalence of type 2 diabetes mellitus (T2DM) and cardiovascular risk factors (8). On the other hand, due to inadequate efficiency complications for this proceeding disorder, researchers have focused attention on the use of medicinal plants. Some of these plants and their active constituents are effective in the treatment of MetS, including *Nigella sativa* (9), *Camellia sinensis *(10), *Silybum marianum* (11), *Persea americana* (12), *Crocus sativus* L. (13), *Garcinia mangostana* (14), *Capsicum annuum *(15), *Vitis vinifera* (16), *Berberis vulgaris* (17), rutin (18), and *Solanum melongena* (19). 

**Figure 1:**
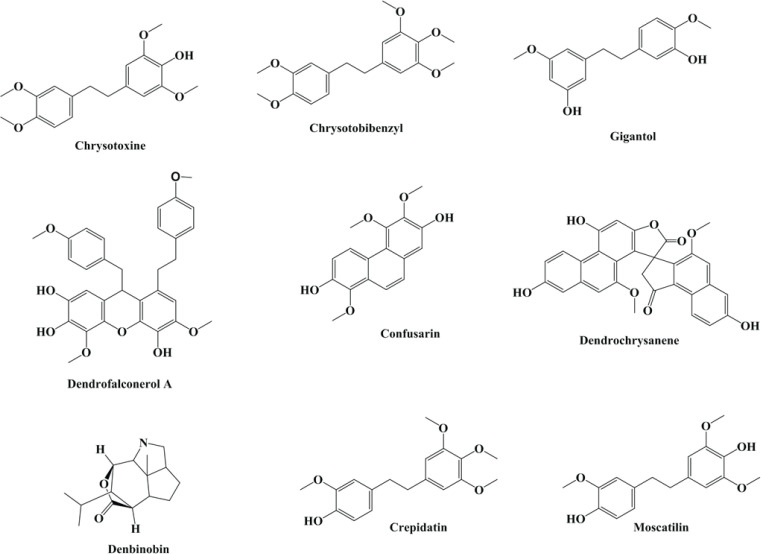
The bioactive constituents of *Dendrobium* include gigantol, moscatilin, dendrofalconerol A, dendrochrysanene and denbonobin

**Figure 2:**
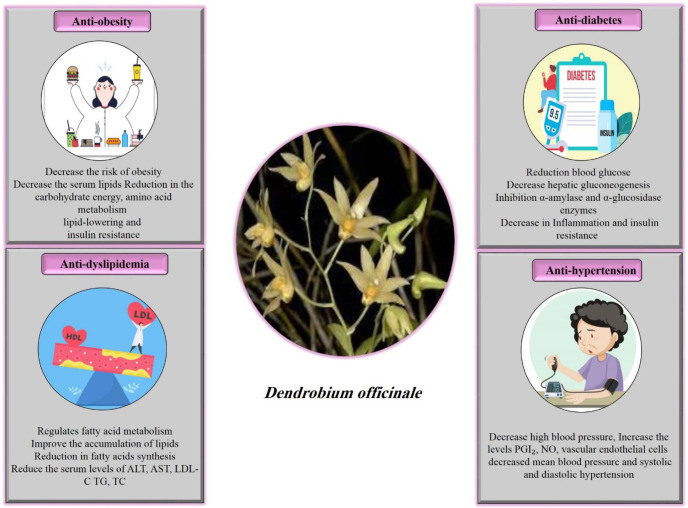
Schematic mechanistic description of *Dendrobium officinale* effects on metabolic syndrome

Orchidaceae is a diverse family of flowering plants with about 27,800 species (20). Among several orchid species, *Dendrobium* is identified as the most popular and largest orchid genera (21). The genus *Dendrobium* contains almost 1400 species, which are found in Australia, the Pacific Islands, and Asia (22). Also, there are morphological differences among various species of *Dendrobium*. In some species, flowers appear in pairs or threes on a peduncle along the entire length of the pseudobulbs, with caduceus-like leaves. In some groups, small flowers arise from leaf axils, while the flowers are paired up or alternately closely set to form pendants or erect thyrses in another species (23). Moreover, the *Dendrobium* genus, approximately 74 species of which have been distributed in China’s tropical and subtropical regions, has been used as a herbal medicine in the treatment of symptoms of diseases such as increasing the production of body fluids, nourishing the stomach, reducing throat inflammation, and improving eyesight for thousands of years (24, 25). Previous research projects have shown that *Dendrobium* contains components such as alkaloids, flavonoids, bibenzyls, terpenes, phenanthrenes, steroids, lignans, and polysaccharides that have important pharmacological properties (26, 27). According to studies (28, 29), the most relevant phytochemical elements of *Dendrobium* species in metabolic syndrome include polysaccharides, alkaloids, and polyphenols. The bioactive constituents of *Dendrobium* include gigantol, moscatilin, dendrofalconerol A, dendrochrysanene, cripidatin, confusarin, denbinobin, and chrysotobibenzyl are shown in Figure 1. According to previous evidence, *Dendrobium* showed various pharmacological properties, including anti-inflammatory (30, 31), anti-fungal (32), antimicrobial (33), anti-oxidant (34-36), antidiabetic (37, 38), hepatoprotective (39, 40), anti-hyperglycemic (29, 41, 42), anti-insulin resistance (43, 44), anti-hypertensive (45, 46), and anticancer (47, 48) (Table 1). Also, the effect of *D. officinale* on MetS has been determined in Figure 2. Regarding the data in hand, this review aims to highlight the beneficial and potential properties of *Dendrobium *on MetS and its complications.

***Methods***

The information was collected by searching PubMed, the Web of Science, Google Scholar, and Scopus. These data have been gathered in the English language, with no time limitation. All types of related books, articles, and abstracts were included. The search keywords included “anti-hyperglycemic”, “antihypertensive”, “antidiabetic”, “atherosclerosis”, “obesity”, “blood pressure”, “blood glucose”, “*Dendrobium*”, “diabetes”, “dyslipidemia”, “high cholesterol”, “hypercholesterolemia”, “hyperglycemic”, “hyperlipidemia”, “body mass index”, “waist circumference”, “hypertension”, hypertriglyceridemia”, “hypoglycemic”, “hypotensive”, “insulin”, “insulin-resistance”, “metabolic syndrome”, “triglyceride”, “atherogenic” and “ weight loss”.

***Effect of Dendrobium on diabetes***

Diabetes mellitus, as a significant global health threat, is a cluster of MetS identified with hyperglycemia and destruction of cellular resistance to both insulin and insulin secretion (49). Insulin resistance is characterized by a reduction of the appropriate response to insulin stimulation, glycogen synthesis, and lipid oxidation. Insulin resistance also has a critical impact on the pathogenesis of MetS, including T2DM and obesity (50, 51). Diabetes is a leading cause of macro-vascular and microvascular complications and dramatically increases the risk for nephropathy, retinopathy neuropathy, and cardiovascular diseases (CVDs) (52, 53). Hyperglycemia is closely related to the excess generation of reactive oxygen species (ROS) and oxidative stress which can up-regulate the levels of inflammatory factors including tumor necrosis factor-alpha (TNF-α), interleukin-6 (IL-6) and reduces the level of interleukin 10 (IL-10). Moreover, TNF-α induces insulin resistance and hyperlipidemia by activating nuclear factor-κB (NFĸB) (54). Different molecular mechanisms such as α-glucosidase activity associated with hyperglycemia have been identified. Inhibition of this enzyme can significantly delay carbohydrate absorption and decrease the postprandial elevation of blood glucose levels after meals (55). Cyclic adenosine monophosphate protein kinase A (cAMP/PKA) can regulate glucose homeostasis in several processes, including glucose uptake, glucagon and insulin secretion, gluconeogenesis, glycogenesis, and glycogen degradation (56). The roles of peroxisome proliferator-activated receptor-α (PPAR-α) in mediating diabetes-related molecular events and increasing insulin secretion via regulating fat and β-oxidation of adipocytes in islet β cells have been explored (57). Also, the glucagon-like peptide-1 (GLP-1) secreted by intestinal L cells participates in promoting insulin release and inhibiting glucagon secretion (58). 

In some studies, the antidiabetic properties of different species of *Dendrobium*, including *D. officinale*, *D. huoshanense*, *D. loddigesii*, *D. officinale*, *D. candidum*, *D. formosum *Roxb. ex Lind, *D. mixture*, *D.* *gibsonii*, *D. moniliforme,* and *D. nobile *Lindl. have been reported and will be discussed in the following.

***In vitro studies***

In a study, the mechanism of hypoglycemic effects of *D. tortile *Lindl. was determined *in vitro* by its α-glucosidase inhibitory activity. In this study, the ethyl acetate extract from the whole *D. tortile *Lindl. resulted in the isolation of compounds such as dendrofalconerol A, which at the concentration of 200 µg/ml, similar to acarbose (antidiabetic medicine) showed strong α-glucosidase inhibition (59). Inhibitory impacts on α-amylase and α-glucosidase enzyme activities have been shown in another study. *D. officinale* constituents exhibited an antidiabetic effect by inhibition of radical scavenging response, α-glucosidase, and α-amylase. Among various compounds that were identified from the crude extract of stem of *D. officinale*, 3,4-dihydroxy-4, 5-dimethoxybibenzyl reported as an α-glucosidase, an α-amylase inhibitor and radical scavenging agent for the first time (60). In another study, the hypoglycemic effect of a shihunine-rich extract of a *D. loddigesii* was investigated on 3T3-L1 cells. In this research, insulin resistance was induced in 3T3-L1 adipocytes by dexamethasone which resulted in decreased glucose uptake. *D. loddigesii* (1.02, 2.03, 4.06, 8.12, and 16.25 µg/ml) treatment significantly raised glucose uptake while reducing the raised level of insulin resistance in 3T3-L1 cells (61). *D. devonianum* and its constitutes including A flavonol glycoside (5-hydroxy-3-methoxy-flavone-7-O-(β-D-apiosyl-(1-6) -βD-glucoside), as well as gigantol showed hypoglycemic effects via inhibiting of the α-glucosidase enzyme with the inhibition rate of 43.4% and 36.7%, respectively (62). In an *in vitro* model, the various concentrations of polysaccharides from *D. officinale* (100, 200, and 400 μg/ml) ameliorated glucose metabolism by regulating the phosphatidylinositol 3 kinase/protein kinase B (PI3K/AKT) signaling pathway which shows an important impact on glycogen synthesis and glucose metabolism in IR HepG2 cells. Besides, the expression of p-PI3K, PI3K, p-Akt, Akt, p-IRS1, p-IR-β, IR-β, and IRS1 in IR HepG2 cells was reduced compared to the control group. By treatment of polysaccharides from *D. officinale* for 24 h, the expression of these proteins significantly increased (63). Two polysaccharides from the stems of *D. officinale* were isolated, and their hypoglycemic activity was evaluated in the murine enteroendocrine cell line (STC-1). In this study, the polysaccharides from *D. officinale* at various concentrations (0, 0.2, 2, 20, 200, and 2000 μg/ml) showed a significant hypoglycemic effect by inducing glucagon-like peptide-1 (GLP-1) secretion in STC-1 cells (64). In a study, a methanol extract from the whole plant of *D. formosum *Roxb. ex Lindl. resulted in the isolation and identification of various compounds, such as moscatilin which at the concentration of 100 µg/ml concerning glucose-uptake stimulation effects, showed greater activity than insulin on L6 myotubes. Also, the glucose uptake stimulation effect was shown at a non-toxic concentration of lusianthridin (1 µg/ml) on L6 myotubes cells. Moreover, methoxy-7-hydroxy-9,10-dihydro-1,4-phenanthrenequinone (50 µg/m) reduced hyperglycemia via inhibiting both pancreatic lipase and α-glucosidase enzymes (65). Inhibitory effects on α-glucosidase enzyme activities have been observed in another study. In this experiment new compound, dendrogibsol, which was isolated from the whole plant of *D.* *gibsonii* exhibited potent α-glucosidase inhibitory activity at the dose of 100 µg/ml as compared with the positive control acarbose (66). To evaluate the antidiabetic and anti-oxidant effects of polysaccharides from *D.* *chrysotoxum *Lindl., an *in vitro* study was performed on mouse splenocytes and Jurkat cells (MCF-7). In this experiment, polysaccharides were isolated from the stem of *D. chrysotoxum* Lindl. and showed potent anti-oxidant and hypoglycemic effects in Jurkat cells. Since the anti-oxidant potential of polysaccharides plays an effective role in preventing the development of diabetes, the beneficial effect of *D. chrysotoxum *Lindl. polysaccharides in treating diabetic patients may be related to their anti-oxidant property (29). In another study, the polysaccharides from *D. officinale* exhibited hypoglycemic activity by inhibiting hepatic gluconeogenesis, glycogen degradation, and ameliorating the liver glucose metabolism in diabetic mice (67).

***In vivo studies***

To assess the hypoglycemic effects of the polysaccharide GXG, which was extracted from the stems of *D. huoshanense*, the T2DM mouse model was established. In this study, intragastric administration of the polysaccharide GXG (50 mg/kg/day and 200 mg/kg/day for 5 weeks) showed hypoglycemic effects via regulating glucose homeostasis in T2DM mice. On the other hand, *D. huoshanense* polysaccharide GXG could normalize hyperglycemia and increase the number of β-cells by reducing β-cells apoptosis in the pancreatic islets. Furthermore, it was suggested that GXG may protect against T2DM via regulating the insulin signaling pathway and multiple steps of the PI3K/AKT action in streptozotocin (STZ) and a high-fat diet (HFD) treated mice (25). 

In another experiment, the antidiabetic activity of the rich-polyphenol extract of *D. loddigesii* (25, 50, and 100 mg/kg, gavage, 8 weeks) was evaluated in diabetic db/db mice. These results showed that *D. loddigesii* (100 mg/kg) demonstrated a decreasing impact on blood sugar levels and insulin resistance in diabetic mice. Also, *D. loddigesii* and its phenols components showed anti-inflammatory properties via reducing the levels of IL-6 and TNF-α proteins and anti-oxidant effects by elevating the levels of biochemical enzymes such as catalase (CAT), superoxide dismutase (SOD), as well as glutathione (GSH) (68). The antidiabetic effects of polysaccharides from *D. officinale* stem (20, 40, 80, and 160 mg/kg for 4 weeks) on T2DM rats have been verified in another study. Blood glucose, the serum level of insulin, and glycated serum protein decreased. Polysaccharides from *D. officinale* may activate the anti-oxidant response, which is important for alleviating liver metabolic syndrome and lipid peroxidation (37). Liu *et al.* investigated the antidiabetic effects of polysaccharides from *D. officinale* and observed that an intraperitoneal administration of the ethanolic extract of the stem (100, 200, and 400 mg/kg, 4 weeks) to STZ-HFD-induced diabetic mice ameliorated hyperglycemia and hepatic glucose metabolism through regulating the liver-glycogen structure and glucagon-mediated signaling pathways. *D. officinale* showed an increase in liver glycogen synthesis, suppressed hepatic gluconeogenesis, and decreased breakdown of glycogen. The results suggested that the regulatory mechanism of these effects may be regulating hepatic glycogen metabolism via the cyclic adenosine monophosphate protein kinase A (cAMP-PKA) signaling pathway in HFD/STZ-induced T2DM mice (67). 

The hypoglycemic properties of water-soluble *D. officinale* from the dry stem (75, 150, and 300 mg/kg, 12 weeks, *IP*) were investigated in diabetic mice. *D. officinale* showed a decrease in the amount of serum fasting insulin (FINS) in mice with diabetic cardiomyopathy. It also increased the expression of PPAR-α and decreased the expression of transforming growth factor-β1 (TGF-β1). This finding suggested that *D. officinale* ameliorated HFD/STZ-induced diabetic cardiomyopathy. A significant decrease in phosphorylation of JNK, an increase in phosphorylation of insulin receptor substrate 1 (p-IRS1), and E-cadherin, known as the epithelial cadherin, by *D. officinale* were observed. Moreover, it was suggested that the possible mechanism for *D. officinale* extract function may be in connection with the activation of the PPAR-α/c-Jun N-terminal kinase (JNK) pathway (69). Inflammation and insulin resistance are mediated by the inhibition of serine phosphorylation of IRS-1 by JNK (70). Chang et al. investigated the functional antidiabetic role of *D. candidum* (0.2, 0.4, and 0.8 g/kg, two weeks, *IP*) on kidneys in diabetic rats. In this study, D*. candidum* played a reno-protective role against diabetic conditions by inhibiting the expression of biological factors including connective tissue growth factor (CTGF), glucose transporter-1 (GLUT-1), and vascular endothelial growth factor (VEGF). Also, *D. candidum* demonstrated important antidiabetic effects under diabetic problems via decreasing the levels of clinical prognosis factors for kidney function such as urea, serum creatinine, and urea nitrogen in blood (71). 

From data obtained in a study, *D. mixture* (12 g/kg, 8 weeks, gavage) significantly decreased the fasting blood glucose (FBG) and improved diabetic nephropathy via reducing the expression transforming growth factor-β1 (TGF-β1)/Smads signal transduction pathway in db/db mice (72). The hypoglycemic effects of *D. officinale *Kimura et Migo in treating diabetic nephropathy were evaluated in another study. According to these results, *D. officinale* (5 and 10 ml/kg, intragastric, for 4 weeks) demonstrated therapeutic potential effects via reducing the activation of FBG, FINS, insulin-resistant, and toll-like receptors (TLRs) in diabetic rats induced by STZ (43). The anti-diabetic activity of an aqueous extract from *D. officinale *Kimura & Migo was investigated in an experiment. In this study, high-dose water extract of *D. officinale* (700 mg/kg, 2 weeks, IP) demonstrated a considerable reduction in random blood glucose levels, while no significant difference was found in the low-dose water extract (350 mg/kg, 2 weeks, IP) group. However, this plant increased the FINS serum in all doses (73). In diabetic rats, exposure to *D. mixture* at the dose of (17.2 g/kg/day, 12 weeks, IP) caused a reduction in FBG, aspartate transaminase (AST) glycosylated serum protein (GSP), and alanine transaminase (ALT). A positive correlation between increased levels of ALT and decreased insulin sensitivity in the liver can be used to predict the development of T2DM. In this study, *D. mixture* decreased the expression of proteins such as glucose‐6‐phosphatase (G6Pase), phosphoenolpyruvate carboxykinase (PEPCK), and signaling molecule Forkhead box O (FoxO1) by increasing the activity of PI3K/ Akt, thereby regulated gluconeogenesis and glucose metabolism under diabetic condition (74). Another study revealed the beneficial hypoglycemic effects of polysaccharides from *D. moniliforme* (100 and 200 mg/kg, ig) on experimental diabetic mice. *D. moniliforme* extract significantly decreased the serum glucose level and elevated glucose tolerance in adrenalin and alloxan-induced diabetic mice (75). 

The hypoglycemic properties of the extracts from *D. nobile *Lindl. (10-80 mg/kg, 8 days, PO) were investigated in hyperglycemic mice induced by adrenalin. Polysaccharides and alkaloids in *D. nobile *Lindl. extract induced a reduction in the level of blood sugar in experimental mice (76). study by Zhang et al. indicated that oral administration of *D. fimbriatum* extracts at the doses of 100 and 200 mg/kg (orally, 2 to 5 weeks) significantly ameliorated diabetes symptoms via inhibiting the inflammatory factors such as cytokines IL-1β and TNF-α and preventing islet cell apoptosis in diabetic rats. This article proposed that regulatory mechanisms of this effect may regulate diabetes and its complications by preventing β-cells apoptosis and decreasing liver lipid accumulation (77). In another study, *D. chrysotoxum* Lindl. ameliorated diabetic retinopathy and retinal inflammation by inhibiting the NF-𝜅B signaling pathway. In this study, *D. chrysotoxum *Lindl. (30 and 300 mg/kg, orally, 4 weeks) could decrease retinal mRNA expressions of intercellular adhesion molecule-1 (ICAM-1) and the serum levels of inflammatory markers such as TNF-α, IL-6, and IL-1β by inhibiting NF-𝜅B activation in diabetic rats. NF-𝜅B family regulates inflammatory responses through the activity of cytokines such as IL-1β and TNF-α. *D. chrysotoxum *Lindl. also reversed the expression of tight junction proteins (including occludin and claudin-1) and alleviated the increased p65, inhibitor of nuclear factor kappa B (IκB), and IκB kinase (IKK) in diabetic rats. Therefore, *D. chrysotoxum *Lindl. ameliorated an inflammation of the retina by inhibiting the NF-𝜅B signaling pathway in STZ-induced diabetic rats (78). The anti-diabetic effect of polysaccharides from *D. chrysotoxum *Lindl. (200 and 500 mg/kg, orally, for 1 week) on alloxan-induced diabetic mice was evaluated. This compound decreased the level of blood glucose in all of the doses. Also, *D. chrysotoxum *Lindl. showed anti-oxidant activity by inhibiting hydroxyl radicals (OH)-mediated deoxyribose degradation and superoxide anions (O2^• −^) against glucose oxidase mediated cytotoxicity in Jurkat cells. Thus, these results suggest that *D. chrysotoxum* may exert hypoglycemic effects through anti-oxidant activity (29). 

In another study, administration of polysaccharides from *D. huoshanense* (50, 100, and 200 mg/kg, IP) decreased blood sugar levels in diabetic cataract rats. These compounds improved cataract diabetes by inhibiting the oxidation pathway, which down-regulated inducible nitric oxide synthase (iNOS) gene expression and advanced glycation end products (AGEs) formation (79). 

An *in vivo* study demonstrated the differences in hypoglycemic effects among various *Dendrobium* species on the metabolic syndrome. In these results, the oral administration (50, 100, and 200 mg/kg) of polysaccharides from* D. officinale*, *D. nobile*, and *D. huoshanense *indicated significant anti-diabetic effects. *D. officinale*, *D. nobile, *and *D. huoshanense* but not *D. chrysotoxum* showed anti-diabetic activity in the decreasing order of *D. huoshanense *> *D. nobile *> *D. officinale *> *D. chrysotoxum *(80).

According to the above-mentioned results, the various species of *Dendrobium* at different doses (high doses and low doses) and times of exposure significantly decreased blood sugar levels through various mechanisms in diabetic study models. It seems inhibition of the NF-𝜅B signaling pathway, decreasing hydroxyl radicals, and increasing anti-oxidant activity are some of the most important mechanisms to manage the antidiabetic effects of these plants (Table 2).

***Effect of Dendrobium on serum lipid profile***

Hyperlipidemia normally refers to high levels of cholesterol, TG, and changes in lipoprotein patterns within the human body (81). Hyperlipidemia is also described as a lipoprotein metabolism disorder that leads to atherosclerosis, coronary artery disease and metabolic syndrome. Additionally, hyperlipidemia is known as an established risk factor for CVD disorder which is the leading cause of mortality worldwide (82). Among various mechanisms in lipid metabolism, PPAR-α has a crucial impact on lipid metabolic processes and accelerates β-oxidation of adipocytes in islet β cells (83). The JNK signaling pathway is also known to contribute to regulating the PPAR-α -FGF21 hormone (84). Fatty acid β-oxidation is another pathway for lipid metabolism that occurs in both mitochondria by carnitine palmitoyltransferase 1 (Cpt1) and peroxisomes by Acyl-CoA Oxidase 1 (Acox1) (85). Among the various herbal medicine compounds, *Dendrobium* species have been shown to have potent anti-dyslipidemia effects in different experimental models, which are discussed in the next sections.

***In vitro studies***

In a study, the stems of *D. loddigesii* (2.03, 4.06, 8.12, and 16.25 µg/ml) significantly decreased the intracellular accumulation of fat droplets and TG as well as promoted the 2-[N-(7-nitrobenz-2-oxa-1,3-diazol-4-yl) amino]-2 deoxyglucose (2-NBDG) uptake of 3T3-L1 cells. *D. loddigesii *also revealed its lipid-lowering effect by increasing in p-AMPK and GLUT-4 levels in the adipose tissue and a rise in expression levels of p-AMPK and PPAR-γ in the liver tissue. Thus, it showed the hypolipidemic effects by up-regulating the expression of proteins such as PPAR, p-MAPK, and GLUT4 (61). 

The inhibitory effect of a polysaccharide compound (DHP1A) obtained from *D. huoshanense* (0.5, 0.1, 1.5, and 2.0 mg/ml) on the FeCl_2_-induced lipid peroxidation in mice’s liver was examined *in vitro*. According to these data, a variety of mechanisms, such as an increase in the levels of free radical scavenging including glutathione peroxidase (GPx), CAT, SOD, as well as GSH are involved in the reduced activity of dyslipidemia and MDA content. The anti-oxidant response of DHP1A was more remarkable than that of dextran under the same conditions; this may be due to its unique structural features. Also, it showed an inhibitory effect on the FeCl_2_-induced lipid peroxidation. Therefore, anti-lipid peroxidation essentially played a role in the anti-oxidant mechanisms of DHP1A (86). 

***In vivo studies***

The anti-hyperlipidemia effects of water-soluble extracts of *D. officinale* (75, 150, and 300 mg/kg, 12 weeks, IP) have been studied in diabetic and HFD rats. In this study, *D. officinale* **i**ncreased fatty acid metabolism, significantly decreased LDL-C, TC, FINS, and TG, and increased HDL-C levels in the serum. The possible mechanism of these effects may be associated with the expression activation of PPAR-α and a decrease in the expression of p-JNK proteins (69). 

In another experiment, ultrafine *D. officinale* powder (0.6 g/kg per day, 3 weeks, PO) regulated fatty acid metabolism to improve the accumulation of lipids in a mouse model of progressive nonalcoholic fatty liver disease (NAFLD) on the high-sucrose, high-fat (HSHF) diet. The positive correlation between fatty acid metabolism disorders and the development of NAFLD in liver injury has been confirmed. According to the results, *D. officinale* powder ameliorated liver lipid metabolism in NAFLD mice by reducing the levels of hepatic lipids, including TC, TG, triacylglycerol (TAGs), AST, ALT, and free fatty acid (FFA). Reduction in fatty acids synthesis, uptake, and desaturation, and promotion of FA β-oxidation were also observed after treatment with *D. officinale* powder (87). 

The results of a study on hyperlipidemic rats suggested that an aqueous extract from *D. officinal* showed an anti-hyperlipidemic effect at doses of 0.25, 0.5, or 1 g/kg by reducing the serum levels of ALT, AST, LDL-C, TG, and TC, as well as a significant elevation in the serum concentrations of HDL-C. Moreover, *D. officinal* inhibited the formation and development of high lipid profiles and enhanced anti-oxidant capacity in hyperlipidemic rats (88). 

Various studies have shown that hypercholesterolemia (HCD) is a variable risk factor that forms atherosclerosis plaques by increasing levels of lipid profiles. In an experiment, *D. huoshanense* polysaccharide inhibited plaque formation by decreasing lipid accumulation in the blood vessels of the AS zebrafish. Moreover, it showed a significant decrease in the levels of ROS, TC, TG, and malondialdehyde (MDA) in zebrafish (89). 

In another study, the hepatoprotective effect of different extracts (water extract, alcohol-soluble extract, alcohol-insoluble extract, and crude extract) of *D. huoshanense* was investigated on sub-acute alcohol-induced hepatic failure in mice. After administration of *D huoshanense* for 30 days, serum concentrations of AST, ALT, alkaline phosphatase (ALP), LDL-C, TC, and TG significantly decreased, and the activation of enzymes such as SOD, alcohol dehydrogenase (ADH), acetaldehyde dehydrogenase (ALDH) and glutathione (GSH-Px) in the liver increased. In this study, freeze-dried *D. huoshanense*, its water extract, and its alcohol-insoluble extract showed better protective effects than that of the alcohol-soluble extract against alcohol-induced liver damage and steatosis, but these effects were lower than those of crude polysaccharides (90). 

In a study, *D. nobile *Lindl. alkaloids (10–80 mg/kg, 8 days, PO) reduced hyperlipidemia by activating Acox1 and Cpt1a genes in mice fed with HFD. Furthermore, *D. nobile *Lindl. alkaloids improved metabolic liver diseases in animals via upregulating adipose triglyceride lipase (ATGL/Pnpla2), which was mediated by the activation of PPAR-α. Decreasing the expression of sterol regulatory element-binding protein 1 (Srebp1), as a mechanism of lipid-lowering, was another beneficial effect of *D. nobile *Lindl. alkaloids in the metabolic syndrome (76).

The other experiments carried out to investigate the effect of the rich-polyphenol extract of *D. loddigesii *on lipid serum in diabetic mice confirmed the above-mentioned activities. In this study, *D. loddigesii* (25, 50, and 100 mg/kg, 8 weeks, gavage) showed a significant reduction in the levels of LDL-C, TC, and TG while increasing HDL-C blood serum levels (68).

The results of a study on *D. mixture* (17.2 g/kg/day, 12 weeks, IP) in HFD and highsugar diet rats demonstrated a significant reduction in serum ALT, AST, and GSP levels. Moreover, a reduction in lipid profiles such as LDL-C, TC, and very-low-density lipoprotein cholesterol (VLDL-C) was noted, while the serum level of HDL-C increased (74). 

The hypolipidemic effects of *D. candidum *Wall. ex Lindl. (200 or 400 mg/kg, 6 weeks, i.g.) were evaluated on mice with HFD. *D. candidum* decreased lipid accumulation and maintained lipid metabolism and glucose homeostasis. Accordingly, *D. candidum* significantly inhibited the hepatic inflammasome activation of the NLR family pyrin domain containing 3 (NLRP3) and elevated the expression of gluconeogenesis-related genes and lipid metabolism in HFD mice. The results indicated that this plant may be a useful therapeutic strategy against NAFLD damage (40). 

Different spices of *Dendrobium* demonstrated a significant reduction in serum levels of lipid profiles. These findings are supported by various studies that *Dendrobium* decreased the levels of LDL-C, TC, and TG while it increased HDL-C blood serum levels. In addition, *Dendrobium* species through other mechanisms such as anti-oxidant activity, increase in expression of PPAR-α, and decrease in expression of p-JNK proteins showed hypolipidemic effects at the different doses and times of exposure (Table 3). 

***Effect of Dendrobium on high blood pressure***

Hypertension is a very common condition that leads to an increase in the incidence of CVD, myocardial infarction, stroke, ischemia, and atherosclerosis (91-93). This phenomenon is a progressive medical condition that is prevalent in the world and causes premature mortalities (94). Blood pressure leads to increased post-cardiac load and cardiovascular dysfunction, resulting in compensatory cardiac hypertrophy (95). Although antihypertensive therapy has been used for many years to decrease the risk of morbidity and mortality, the side effects of these drugs, including calcium channel inhibitors and angiotensin-converting enzyme blockers tend to decrease medication adherence. Therefore, natural compounds with fewer side effects and potential anti-hypertensive therapeutic values are used to treat elevated blood pressure (96). Several studies have demonstrated the anti-hypertension effect of various species of *Dendrobium* via different mechanisms, which are discussed in the next sections. 

***In vitro studies***

In an *in vitro* model, the aqueous extract of *D. candidum* (2 mg/ml) improved cardiac hypertrophy by regulating the extracellular signal-regulated kinase (ERK) signaling pathway in the rat cardiac myocyte H9c2 cells incubated with isoproterenol. According to the results, *D. candidum* lowered the up-regulated mRNA expression levels of brain natriuretic peptide (BNP) and ANP induced by isoproterenol by inhibition of the ERK pathway (97).

***In vivo studies***

The beneficial effects of the *Dendrobium* compound in preventing blood pressure were investigated using hypertensive rats. This agent was mixed with diet and fed to the rats for 4 weeks. Results showed that the *D. candidum* compound (1.65, 3.30, and 5.00 g/kg) significantly reduced the blood pressure and exhibited anti-hypertensive activity by activating PI3K/AKT/endothelial nitric oxide synthase (eNOS) signaling pathways and increasing the levels of serum nitric oxide (NO) generation. Besides, this plant significantly prevented hypertension and vascular dysfunction in hypertensive rats by inhibiting the secretion of ICAM-1 and the levels of serum endothelin-1 (ET-1) (98).

The antihypertensive effect of *D. officinale* flos was evaluated on hypertensive rats caused by high-fat and glucose compound alcohol. In this study, *D. officinale* flos (3, 1 g/kg for 6 weeks) normalized systolic blood pressure, mean arterial pressure, and improved vascular diastolic dysfunction. Also, this plant reduced the plasma content of ET-1, thromboxane B₂ (TXB₂), and thickening of the thoracic aorta, while the levels of prostacyclin (PGI₂), NO, and the activities of vascular endothelial cells increased (99). 

In a study, *D. officinale* granules improved hypertension in hypertensive rats induced by long-term alcohol. This agent was fed to the rats for 32 weeks and not only decreased hypertension (mean blood pressure and systolic and diastolic hypertension) but also showed notable improvement in the lipid profile besides liver and kidney injuries (100).

In an experiment, the aqueous extracts of *D. candidum* (0.13 and 0.78 g/kg, orally, for 4 weeks) alleviated cardiac hypertrophy and improved heart function in isoproterenol-induced cardiac hypertrophy models through inhibition of the ERK signaling pathway. It is worth noting that *D. candidum* prevented cardiac hypertrophy by reducing the left ventricular systolic pressure (LVSP), heart-to-body weight ratio (HW/BW), left ventricular/tibia length (LV/TL), and atrial natriuretic peptide (ANP) (97). In a study, *D. officinale *Kimura et Migo (5 and 10 ml/kg, intragastric< for 4 weeks) significantly reduced hypertension in rats with diabetic nephropathy (43). In another experiment, the antihypertensive effects of *D. officinale* in another experiment were investigated. In this study, *D. officinale* showed protective effects against high blood pressure by triggering the enteric-origin short-chain fatty acid- G-protein-coupled receptors (SCFA-GPCR43/41) pathway in metabolically hypertensive rats (101).

Therefore, these results suggest that different species of *Dendrobium* such as *D. candidum *and* D. officinale* through inhibition of the ERK signaling, reduction in plasma content of ET-1, TXB₂, and thickening of the thoracic aorta, increase the levels of PGI₂, NO and the activities of vascular endothelial cell demonstrated hypotension effects in experimental models (Table 4). 

***Effect of Dendrobium on obesity***

Obesity is a critical global issue, and its prevalence is increasing in both developed and developing countries (102). It is considered the fifth leading risk factor for mortality according to the World Health Organization (WHO) and greatly increases the risk of chronic diseases including diabetes, CVD, cancer, neurodegenerative diseases, rheumatoid arthritis, and osteoarthritis (103, 104). Obesity is a state of pathological increase in the amount of adipose tissue, which is closely associated with an imbalance between food intake and energy expenditure (105). Disorders in the secretion of adipokines, specific cytokines of adipocytes in the obese state, result in changes in lipid and carbohydrate metabolism and may lead to insulin resistance and diabetes (103). PPAR-γ has been reported to play an important role in the regulation of lipid metabolism in adipocytes through fatty acid trapping (106). Obesity-related diseases may be effectively treated by preventing or treating insulin resistance and abnormal lipid metabolism. Few studies have investigated the use of natural products to treat obesity, such as *Dendrobium*, which has demonstrated promising anti-obesity properties in several reports.

***In vitro studies***

The inhibitory effect of the *D. officinale *polysaccharide (200 μg/ml for 48h) was assessed on palmitic acid-induced insulin resistance in 3T3-L1 adipocytes, C2CL2 myocytes, and hepatocytes. The results showed that *D. officinale* treatment significantly promoted the insulin-stimulated glucose uptake of 3T3-L1 adipocytes and C2C12 myocytes, while the glucose output of hepatocytes decreased. Therefore, it is likely that *D. officinale* is an agent with potential therapeutic or preventive effects against insulin resistance. According to these results, *D. officinale* improved the expression of PPAR-γ in myocytes, adipocytes, and hepatocytes (107).

***In vivo studies***

The protective effect of *D. moniliforme* extract (200 mg/kg, orally, for 9 weeks) on obesity-induced renal damage in HFD mice was investigated. In this study, *D. moniliforme* showed a lipid-lowering effect in HFD-induced obesity in mice. Furthermore, after *D. moniliforme* extract administration, the increased body weight, free fatty acid, TC, LDL-c, and TG levels, and the renal lipid accumulations of HFD-fed mice were also significantly reduced (108).

The bodyweight lowering and hypolipidemic effects of *D. officinale* (150 mg/kg) on obesity-induced HFD mice were investigated for 12 weeks. The results revealed a significant reduction in hepatic lipid accumulation through decreasing serum lipid levels (TG, TC, and LDL-C) and increasing HDL-C content. Furthermore, *D. officinale* improved the risk of obesity-associated abnormal lipid metabolism and insulin resistance by enhancing the expression of PPAR-γ, which acts as a potent therapeutic agent for obesity-associated lipid metabolism disorder and insulin resistance (107). 

The effect of *D. officinale* at a dose of 2.37 g/kg induced by an HFD in mice was investigated for 40 consecutive days. In this study, oral administration of *D. officinale* resulted in a reduction in carbohydrate energy, amino acid metabolism of intestinal mucosal flora, food intake, and body weight gain. Moreover, this extract showed better lipid-lowering properties in female mice than in male mice (109). 

To evaluate the anti-obesity and hypolipidemic effects of tin caulis *Dendrobium *polysaccharide, HFD rats were administered a dose of 500 mg/kg of this plant orally for 8 weeks. In this experiment, *Dendrobium* polysaccharide showed a significant improvement in fat tissue hypertrophy and excessive fatty deposition in the liver and also decreased food consumption and body weight (110).

The hypolipidemic and bodyweight loss effects of a rich-polyphenols extract of *D. loddigesii* on diabetic mice were investigated. The present study demonstrated that *D. loddigesii* (100 mg/kg, for 8 weeks) significantly decreased body weight, blood glucose, and fatty liver in the mice. On the other hand, *D. loddigesii* exhibited a significant reduction in serum lipids (TG, TC, and LDL-C), while the status of anti-oxidant and HDL-C activity increased (68). 

These results are provided in response to the question of whether *Dendrobium* has protective effects on obesity via decreasing LDL-C, TC, FINS, and TG, and growing the HDL-C levels in the serum. Also, *Dendrobium* shows its anti-obesity effects by improving fat tissue hypertrophy and excessive fatty deposition in the liver. Thus, this plant is effective at decreasing symptoms of bodyweight and lipid-lowering, and it can be said with confidence (Table 5).

***Clinical studies***

Although several studies are investigating the protective effects of different species of *Dendrobium*, only a few clinical studies have been conducted. 

In a clinical study, 120 T2 DM patients received *Dendrobium* compound (2 g/day) for 90 days. In this study, *Dendrobium* demonstrated a potent antidiabetic effect on patients via decreasing the levels of fasting FBG, 2 hr postprandial blood glucose (2h BG), and insulin resistance (111). Additionally, the safety and effectiveness of *D. huoshanense* were determined with laboratory and clinical tests. Oral administration of the polysaccharide from *D. huoshanense* (2000-4000mg, 4 weeks) could significantly decrease cytokine levels associated with atopic dermatitis and show beneficial effects on symptoms. No serious adverse effects happened during all 4 weeks of treatment (112). Moreover, the oral effects of *D. candidum* extract (0.5 g/5 ml three times daily) were evaluated in female patients. According to the results, the mentioned dose was safe and promoted the expression of aquaporin-5 (AQP-5) in the labial glands of patients with Sjögren’s syndrome (113). In 2009, clinical research was designed to evaluate the safety of *Runmushu* Oral Liquid (RMS) in postmenopausal patients with xerophthalmia. In this study, *RMS* could show a therapeutic effect and alleviated significantly the eye symptoms in postmenopausal women xerophthalmia groups (114). Additionally, there is ongoing interventional research on the clinical effects of *D. nobile *Lindl., focusing on metabolic syndrome. In the mentioned study, the effective and safe dose range of *D. nobile *Lindl. was predicted at 12 g per day (twice a day, 6 g each time) (115).

***Safety***

In a study to evaluate the minimal lethal dose (LD_10_), the aqueous extract of *D. moniliforme* was administered orally (0, 2500, and 5000 mg/kg) in Sprague-Dawley (SD) rats. According to the previous findings, no adverse effects were observed after oral administration of *D. moniliforme* (5,000 mg/kg or less) in rats. An estimated LD_10_ is over 5,000 mg/kg/body weight (116). In another study, the genetic and oral toxicity of the aqueous extracts of *D. taiseed* Tosnobile (800, 1600, and 2400 mg/kg, 90 days) were investigated in SD rats. No clinical signs of mortality or toxicity were associated with *D. taiseed* Tosnobile administration at any doses during a 90-day sub-chronic investigation in animals (117). Different species of *Dendrobium* have been formulated as eye drops, tablets, and capsules. *D. aurantiacum* is reported to be safe, and no observable signs of toxicity were associated with *D. aurantiacum* eye drops in mice. Also, no irritation or irritability reactions have been reported after single-dose or multiple-dose administration of this plant in rabbits’ and guinea pigs’ eyes or skin (118).

We comprehensively reviewed the protective effects of various species of *Dendrobium*, which are characterized by hypotensive, hypolipidemic, hypoglycemic, and anti-obesity effects in different experimental models. On the other hand, the protective effect of dendrofalconerol A, polyphenols, 3,4-dihydroxy-4′,5-dimethoxybibenzyl, moscatilin, lusianthridin, and dendrogibsol extracted from *Dendrobium* species was reviewed in detail.

This study was limited by the absence of human studies for many suggested effects of *Dendrobium *species.

**Table 1:** Different species of *Dendrobium* and their bioactivities

Ref	Bioactivity	Components		Species name
(37)	Antioxidant, anti-diabetic activities		Polysaccharides	*D. officinale*
(76)	Neuronal protective, anti-diabetes, anti-hyperlipidemia		Alkaloids	*D. nobile*
(112)	Anti-inflammatory activity		Polysaccharide	*D. huoshanense.*
(119)	Inhibition of cataractogenesis, Anti-oxidant activity		Bisbenzyl (gigantol)	*D.* *aurantiacum.*
(120)	Anti-angiogenic activity Anti-platelet aggregation activity		Bibenzyls (moscatilin)	*D. loddigesii*
(121)	Antiplatelet aggregation activity		Bibenzyl trigonopols A	*D. trigonopu* ***s***
(122)	Anti-inflammatory activity, Anti-oxidant		Phenanthrenes (dendrochrysanene)	*D. chrysanthum*
(123)	Anti-cancer		Bisbenzyl (Dendrofalconerol A)	*D. falconer*
(124)	Anti-platelet aggregation activity		Dendroflorin) Fluorenones)	*D. densiflorum*
(30)	Anti-inflammatory activity		Phenanthrene	*D. denneanum*
(125)	Cytotoxic, Antimigratory		Bisbenzyl (gigantol, moscatilin)	*D. brymerianum*
(126)	Anti-oxidant activity		Polysaccharide	*D. denneanum*

**Table 2.** Anti-diabetic effects of different species of *Dendrobium*

Ref	Mechanisms	Results	Dosage mg/kg/day) /Study model	Active constituents/type of extract	Species of* Dendrobium*
*In vivo *studies					
(25)	Increase the number of β-cells/ Regulate the insulin signaling pathway/ phosphatidylinositol 3kinase/protein kinase B (PI3K/AKT) action	Normalize hyperglycemia	50 mg/kg/day and 200 mg/kg/day, 5 weeks/On to STZ-HFD induced diabetic mice	Polysaccharides	*D. huoshanense*
(68)	Antioxidant property	↓Blood glucose ↓Insulin resistance ↑ SOD, CAT, GSH	25, 50, 100 mg/kg, gavage, 8 weeks/On diabetic db/db mice	Polyphenols	*D. loddigesii*
(37)	Antioxidant	↓Blood glucose	20, 40, 80, and 160 mg/kg, 8 weeks/On T2DM rats	Polysaccharides	*D. officinale*
(67)	Cyclic adenosine monophosphate protein kinase A (cAMP-PKA) signaling pathway	Ameliorate hyperglycemia and hepatic glucose metabolism Regulate liver-glycogen structure and glucagon-mediated signaling pathways Increase hepatic glycogen synthesis Suppressed hepatic gluconeogenesis and glycogen degradation	100, 200, and 400 mg/kg, 4 weeks/ On STZ-HFD induced diabetic mice	Polysaccharides	*D. officinale*
(69)	Activation of the PPAR-α/ c-Jun N-terminal kinase (JNK) pathway	Decrease in phosphorylation of JNK increase in p-IRS1and E-cadherin,	75, 150, 300 mg/kg, i.p. 12 weeks/ On HFD/STZ-induced diabetic cardiomyopathy mice	-	*D. officinale*
(71)	Inhibition the expression of connective CTGF, VEGF and GLUT-1.	Normalize hyperglycemia ↓Serum creatinine, ↓blood urea nitrogen ↓ urea	0.2, 0.4, and 0.8 g/kg/ On kidneys in diabetic rats	-	*D. candidum*
(72)	Reduce the expression transforming growth factor-β1 (TGF-β1)/Smads signal transduction pathway	Anti-diabetic effects ↓FBG	(12 g/kg for 8 weeks)/In db/db mice	Not mentioned	*D. mixture*
(43)	Toll-like receptors (TLRs)/ promote insulin secretion of pancreatic islet beta cells	↓FBG ↑FINS ↓insulin-resistant	5 and 10 ml/kg, for 4 weeks/ in diabetic rats induced by STZ	Dried stems	*D. officinale Kimura et Migo*
(73)	Blood glucose level	Anti-diabetic effects	700 mg/kg for 2 weeks	Aqueous extract	*D. officinale Kimura & Migo*
(75)	—	↓Blood glucose ↓Glucose tolerance	100 and 200 mg/kg/ In adrenalin and alloxan-induced diabetic mice	Polysaccharides	*D. moniliforme*
(67)	Prevented β-cells apoptosis and decrease liver lipid accumulation	Ameliorate diabetes symptoms	100 and 200 mg/kg, , 2 to 5 weeks/ In diabetic rats	Extract	*D. fimbriatum*
(74)	Activation of PI3K/ Akt	↓ FBG ↓GSP ↓ ALT ↓ AST Decrease G6Pase, PEPCK and FoxO1	17.2 g/kg/day,12 Weeks/ In diabetic rats	Polysaccharides	*D. mixture*
(78)	Inhibition the NF-𝜅B signaling pathway.	Ameliorate diabetic retinopathy and retinal inflammation in diabetic condition	30 and 300 mg/kg, , 4 weeks/ In STZ-induced diabetic rats	Extract	*D. chrysotoxum Lindl*
(29)	Reduction significantly in blood glucose levels	Hypoglycemic activity	200 and 500 mg/kg, orally for 1 week/ on alloxan-induced diabetic	polysaccharide	*D. chrysotoxum*
*In vitro* studies					
(59)	α-glucosidase inhibitory activity	Hypoglycemic effects	200 µg/ml	Dendrofalconerol A	*D. tortile Lindl*
(60)	Inhibition of α-amylase, α-glucosidase, radical scavenging response.	Hypoglycemic effects		3,4-dihydroxy-4′,5-dimethoxybibenzyl	*D. officinale*
(58)	Increased glucose uptake Reduce the raised level of insulin resistance in 3T3-L1 cells	Anti-diabetic effect	1.02, 2.03, 4.06, 8.12, and 16.25 µg/ml)/3T3-L1 cells	Shihunine-rich extract	*D. loddigesii*
(63)	Regulate the PI3K/AKT signaling pathway	Ameliorate glucose metabolism	100, 200 and 400 μg/ml/ HepG2 cells	Polysaccharides	*D. officinale*
(64)	Induce GLP-1 secretion in STC-1 cells	Hypoglycemic activity	0, 0.2, 2, 20, 200, and 2000 μg/ml/ STC-1 cells	Polysaccharides	*D. officinale*
(65)	Inhibition of both α-glucosidase and pancreatic lipase enzymes	**Reduce hyperglycemia **	100 µg/ml/ L6 myotubes cells 1 µg/ml/ L6 myotubes cells 50µg/ml	Moscatilin Lusianthridin Methoxy-7-hydroxy-9,10-dihydro-1,4-phenanthrenequinone	*D. formosum Roxb. ex Lindl*
(66)	α glucosidase inhibitory activity	Hypoglycemic activity	100 µg/ml	Dendrogibsol	*D. gibsonii*
(29)	Anti-oxidant	Anti-oxidant and hypoglycemic effects	Mouse splenocytes and jurkat cells	Polysaccharides	*D. chrysotoxum Lindl*

STZ: Streptozotocin; HFD: high-fat diet; SOD: superoxide dismutase; CAT: catalase; GSH: glutathione peroxidase; TC: total cholesterol; TG: triglyceride; HDL C: high-density lipoprotein cholesterol; LDL-C: low-density lipoprotein cholesterol; FBG: fasting blood glucose; GSP: glycated serum proteins; ALT: alanine aminotransaminase; AST: aspartate aminotransferase; G6Pase: glucose 6-phosphatase; CTGF: connective tissue growth factor; VEGF: vascular endothelial growth factor; GLUT-1: Glucose transporter1; PEPCK: phosphoenolpyruvate carboxykinase 1; forkhead box O1; NF-𝜅B: nuclear factor-κB; GLP-1: Glucagon-like peptide-1; 2hBG: 2 hr postprandial blood glucose

**Table 3:** Hypolipidemic effects of different species of *Dendrobium*

Ref	Mechanisms	Results	Dosage mg/kg/day) /Study model	Active constituents/ type of extract	Species of Dendrobium
*In vivo *studies					
(69)	Activation expression of PPAR-α and decrease in expression of p-JNK proteins	Increase fatty acid metabolism ↓ TC ↓ TG ↓LDL-C ↓ FINS ↑ HDL-c	75, 150, 300 mg/kg, for12 weeks/ In diabetic and high fat diet rats	Polysaccharides	*D. officinale *
(87)	Reduce the levels of hepatic lipids, including TC, TG, TAGs, AST, ALT and FFA	Ameliorate liver lipid metabolism in NAFLD model Reduction in FA synthesis, uptake, and desaturation and promote FA β-oxidation.	0.6 g/kg per day for 3 weeks/ In a mouse model of progressive NAFLD with the HSHF diet	Polyphenols	*D. officinale*
(88)	Antioxidant and lipid-lowering effects	Anti-hyperlipidemic effect ↓ lipid profile ↓ LDL-C ↓ TC ↓ TG ↓ ALT, AST ↑ HDL-c	0.25g/kg, 0.5g/kg, or 1g/kg/ In hyperlipidemic rats	Polysaccharides	*D. officinale*
(90)	Inhibition plaque formation by decreasing lipid accumulation	↓ HCD ↓ TC ↓ TG ↓ MDA ↓ ROS	In the blood vessels at the atherosclerosis zebrafish	Polysaccharides	*D. huoshanense*
(90)	Antioxidant and hypocholesterolemic activity	↓ LDL-C ↓ VLDL-C ↓ TG, TC ↓ AST, ALT ↑ HDL-c ↑ ADH, ALDH ↑ SOD , GSH-Px	30 days/ On sub-acute alcohol induced liver injury in mice	Water extract, alcohol-soluble extract, alcohol-insoluble extract and crude extract	*D huoshanens*e
(76)	Activation of Acox1 and Cpt1a genes Up regulate The adipose tissue triglyceride lipase (ATGL/Pnpla2) Decrease the expression of Srebp1	Reduce hyperlipidemia	10–80 mg/kg, for 8 days/ in HFD mice	Alkaloids	*D. nobile Lindl. alkaloids*
					
(68)	Lipid-lowering effect	↓ LDL-C ↓ TC ↓ TG ↑ HDL-c	25, 50,100 mg/kg for 8 weeks/ in diabetic mice	rich-polyphenols	*D. loddigesii*
(40)	-	Decrease in lipid accumulation, maintains lipid metabolism NLRP3 inflammasome activation	200 or 400 mg/kg , 6 week/ on HFD diet mice	Active constituents	*D. candidum Wall. ex Lindl*
(74)	Lipid-lowering effect	↓ LDL-C ↓ VLDL-C ↓ TG ↓ ALT ↑ HDL-c	17.2 g/kg/day,12 Weeks/ on HFD and highsugar diet rats	Polysaccharides	*D. mixture*
*In vitro *studies					
(58)	elevate the expression levels of GLUT-4 and p-AMPK in the adipose tissue Increase the expression levels of PPAR_ and p-AMPK in the liver tissue	Lipid-lowering effect Decrease the intracellular accumulation of fat droplets and TG Promote the 2-NBDG uptake	03, 4.06, 8.12, and 16.25 µg/ml/3T3-L1 cells	**-**	*D. loddigesii l*
(86)	Increase in free radical scavenging activity factors (SOD, CAT, GPx, GSH)	Inhibition effect on the FeCl2-induced lipid peroxidation	0.5, 0.1, 1.5 and 2.0 mg/ml	Polysaccharides	*D. huoshanense*

TC: total cholesterol; TG: triglyceride; HDL C: high-density lipoprotein cholesterol; LDL-C: low-density lipoprotein cholesterol; FINS: fasting insulin; ALT: alanine amino transaminase; AST: aspartate aminotransferase; NAFLD: non-alcoholic fatty liver disease; TAG: Triacylglycerol; FFA: free Fatty Acids; HCD: high-cholesterol diet; ROS: reactive oxygen species; VLDL-C: very low-density lipoprotein cholesterol; ALDH: aldehyde dehydrogenase; GLUT-4: glucose transporter 4; AMPK: AMP-activated protein kinase; SOD: superoxide dismutase; GPx: glutathione peroxidase

**Table 4:** Hypotensive effects of different species of *Dendrobium*

Ref	Mechanisms	Results	Dosage mg/kg/day) /Study model	Active constituents/ type of extract	Species of Dendrobium
*In vivo *studies					
(98)	Activation of PI3K/AKT/eNOS signaling pathways ↑NO generation inhibition ICAM-1 and ET-1	Anti-hypertensive activity Reduce hypertension and vascular dysfunction in a hypertensive model	1.65, 3.30, 5.00 g·kg,4 weeks/ on hypertensive SD rats fed with high-sugar, high-fat diet and alcohol	-	compound *Dendrobium*
(45)	Reduction in plasma content of ET-1, TXB₂ and thickening of the thoracic aorta increase the levels PGI₂, NO and the activities of vascular endothelial cells	Normalized systolic blood pressure, mean arterial pressure and improved vascular diastolic dysfunction	3, 1 g kg⁻¹ , 6 weeks/ hypertensive rats induced by high glucose and high fat	-	*D. officinale*
(100)	Decrease blood pressure (systolic blood pressure, diastolic blood pressure and mean blood pressure	Improve hypertension	32 weeks/ hypertensive rats induced by long-term-alcohol	-	*D. officinale*
(99)	Decrease systolic blood pressure and lipid profile	alleviate hypertension and metabolic disorders in metabolic hypertension	4 weeks/ on metabolic hypertensive rats induced by high-sugar, high-fat diet and alcohol	-Ethanol extract	*D. officinale* *granule*
(97)	Inhibition of the ERK signaling	Alleviate heart function and cardiac hypertrophy Reduce LVSP HW/BW, LV/TL and ANP	0.13 and 0.78 g/kg, 4 weeks/ isoproterenol-induced cardiac hypertrophy model	-	*D. candidum*
*In vitro *studies					
(97)	Inhibition of the ERK signaling	Improves cardiac hypertrophy Reduce the up-regulated mRNA levels ANP	2 mg/ml/ rat cardiac myocyte H9c2 cells	**-**	*D. candidum*

NO: nitric oxide; ICAM-1: intercellular adhesion molecule1; ET-1: endothelin 1; TXB₂: thromboxane B₂; PGI₂: prostacyclin; ERK: extracellular-signal-regulated kinase; LVSP: left ventricular systolic pressure; HW/BW: heart to-body weight ratio; LV/TL: left ventricular/tibia length; ANP: atrial natriuretic peptide

**Table 5:** Anti-obesity effects of different species of *Dendrobium*

Ref	Mechanisms	Results	Dosage mg/kg/day) /Study model	Active constituents/	Species of *Dendrobium*
*In vivo *studies					
(108)	lipid-lowering effect	Lipid-lowering effect in HFD-induced obesity Decrease body weight ↓TC ↓ LDL -c, TG Decrease free fatty acid level and renal lipid accumulations	200 mg/kg, 9 weeks/ High fat diet mice	Methanolic extract	*D. moniliforme*
(107)	↓TC ↓ LDL -c, TG ↑HDL Up-regulate the expression of PPAR-γ	Decrease in liver lipid accumulation Improve obesity-associated abnormal lipid metabolism and insulin resistance	150 mg/kg, 12 weeks/ on obesity-induced HFD mice	Polysaccharide	*D. officinale*
(109)	-	Reduction in the carbohydrate energy, amino acid metabolism of intestinal mucosal flora, food intake and the bodyweight gain lipid lowering effect	2.37 g·kg−1, 40 days/High fat diet rats·	Not mentioned	*D. officinale*
(76)	Improve in fat tissue hypertrophy and excessive fatty deposition in the liver	Decrease in food consumption and body weight	500 mg/kg,8 weeks/ High fat diet mice·	Polysaccharide	*D.tin caulis *
(68)	-	Decrease body weight, improve the fatty liver ↓TC ↓ LDL -c, TG ↑HDL-C, antioxidant status	100 mg/kg , 8 weeks/Male obese diabetic mice	polyphenols	*D. loddigesii*
*In vitro* studies					
(107)	Up-regulate the expression of PPAR-γ in adipocytes, myocytes, and hepatocytes	Promote the insulin-stimulated glucose uptake of 3T3-L1 adipocytes and C2C12 myocytes, while the glucose output of hepatocytes decreased.	200 μg/ml for 48h/3T3-L1 adipocytes, C2CL2 myocytes, and hepatocytes	Polysaccharide	*D. Officinale*

HFD: high fat diet; TC: total cholesterol; TG: triglyceride; HDL C: high-density lipoprotein cholesterol; LDL-C: low-density lipoprotein cholesterol; TC: total cholesterol; TG: triglyceride; NO: nitric oxide; PGI2: prostacyclin

## Conclusion

In this review, our team summarized different *in vitro*, *in vivo*, and clinical studies to find out the role of different species of *Dendrobium* and their active constituents on MetS which is known as a global health challenge. According to the experimental studies reported in the literature, the different species of *Dendrobium* can be good candidates for managing MetS complications, including diabetes, hypertension, dyslipidemia, and obesity. These plants regulate dyslipidemia by reducing the levels of LDL-C, TG, and TC and elevating the levels of HDL-C in the blood by various mechanisms, such as anti-oxidant activity, and up-regulating the expression of PPAR-γ and p-AMPK in the liver tissue. *Dendrobium* also decreases hypertension via activating PI3K/AKT/eNOS signaling pathways, NO generation, and inhibition of ICAM-1 and ET-1. Furthermore, the hypoglycemic effects of species of *Dendrobium *can be mediated by various mechanisms, such as increasing the number of β-cells, regulating the insulin signaling pathway, and PI3K/AKT action. Despite the promising findings of several in *vitro* and in *vivo* studies, the lack of human studies about the safety and efficacy of *Dendrobium *is obvious. Hence, further clinical research projects are needed to confirm the effectiveness and safety of these plants as candidates for the treatment of MetS in humans.

## Authors’ Contributions

HH Study conception, design and supervision of the research; MGR Critical revision of the paper, supervision of the research; ZO Preparation of original draft. All authors have agreed to the contents and approved the final version for publication

## Funding

This review received no specific grant from any funding agency in the public, commercial, or not-for-profit sectors.

## Conflicts of Interest

The authors declare that they have no conflicts of interest.
